# Localisation exceptionnelle de condylomes acuminés dans les fosses nasales d’une patiente vivant à Yaoundé, Cameroun: cas clinique et revue de la littérature

**DOI:** 10.11604/pamj.2020.36.349.24918

**Published:** 2020-08-26

**Authors:** Antoine Bola Siafa, Yves Christian Andjock Nkouo, Sandrine Owona, Grâce Nganwa, François Djomou

**Affiliations:** 1 Service Oto-Rhino-Laryngologie et Chirurgie Cervico-Faciale du Centre Hospitalier Universitaire de Yaoundé, Yaoundé, Cameroun,; 2 Département d’Ophtalmologie, d’Oto-Rhino-Laryngologie et de Stomatologie de la Faculté de Médecine et des Sciences Biomédicales de l’Université de Yaoundé I, Yaoundé, Cameroun,; 3 Service Oto-Rhino-Laryngologie et Chirurgie Cervico-Faciale de l’Hôpital Général de Yaoundé, Yaoundé, Cameroun,; 4 Département d’Anatomopathologie de la Faculté de Médecine et des Sciences Pharmaceutiques, Université de Douala, Douala, Cameroun

**Keywords:** Condylomes acuminés, fosses nasales, *human papillomavirus*, Yaoundé, Cameroun, Condylomata acuminata, human papillomavirus, nasal cavity, Yaoundé, Cameroon

## Abstract

Les condylomes acuminés sont des lésions sexuellement transmissibles causées par le human papillomavirus. Ils se localisent surtout sur la sphère ano-génitale et sont exceptionnellement rencontrés dans les fosses nasales. Nous en rapportons un nouveau cas. Une patiente séropositive au VIH et suivie en gynécologie pour récidive de condylomes acuminés vulvaires, nous est adressée pour prise en charge de masses rosées des fosses nasales empêchant la respiration nasale et évoluant depuis plusieurs semaines. L’examen clinique et le scanner suspectent des condylomes, un traitement chirurgical est effectué sous anesthésie générale sous contrôle endoscopique et l’histologie revient en faveur de condylomes acuminés. Les suites postopératoires sont simples et sans récidive à 6 mois postopératoire. La localisation nasale des condylomes acuminés est rare voire exceptionnelle, seule une poignée de cas sont décrits dans la littérature. Le profil est celui d’un adulte jeune séropositif ou non avec notion de comportement sexuel à risque. Plusieurs localisations notamment génitales peuvent être retrouvées et dans ce cas la greffe nasale des lésions pourrait être manuportée. Le traitement chirurgical est efficace et le suivi à long terme est indispensable pour prendre en charge d’éventuelles récidives.

## Abstract (en)

Condylomata acuminata are sexually transmitted lesions caused by human papillomavirus. They mainly occur in the anogenital area and exceptionally in the nasal cavity. We here report a new case of uncommon detection. The study involved a HIV-positive female patient with recurrence of vulvar condylomata acuminata treated in the department of gynecology. She was referred to our department with pink masses in the nasal cavity preventing nasal breathing and evolving over several weeks. Clinical examination and computerized tomography (CT) scan suggested condylomata. Surgical treatment was performed under general anesthesia and endoscopic control; the histological examination showed condylomata acuminata. The postoperative course was simple, with no recurrence after 6 months of follow-up. Condylomata acuminata in the nasal cavity are rare or even exceptional; only very few cases have been described in the literature. They commonly affect young subjects with or without HIV infection, having sexual risk behavior and can occur in several areas, in particular in the genital area. In this case, nasal lesions could result from hand-mediated transfer. Surgical treatment is effective and long-term follow-up is essential to treat possible recurrences.

## Introduction

Les condylomes acuminés vulgairement appelés «crêtes de coq» sont des excroissances exophytiques localisées principalement au niveau de la sphère ano-génitale [1]. Ils sont causés par des virus de type *Human PapillomaVirus* (HPV) et sont à transmission sexuelle [1]. Bien que rare voire exceptionnelle, la localisation des condylomes acuminés dans la muqueuse des fosses nasales est possible du fait de la présence désormais bien établie du HPV dans la quasi-totalité de la muqueuse des voies aériennes supérieures [2]. Nous présentons un cas de localisation de condylomes acuminés dans les fosses nasales chez une jeune femme de 33 ans, prise en charge chirurgicalement. Une revue de la littérature succincte sur cette entité sera ensuite déroulée en insistant sur le diagnostic et la prise en charge.

## Patient et observation

**Figure 1:**
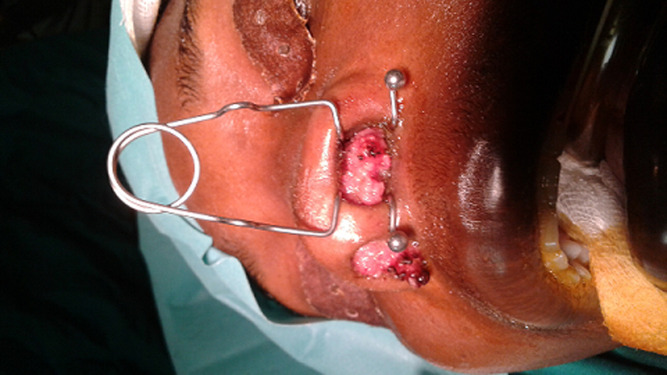
masses aspect crêtes de coq dans le vestibule narinaire en pré opératoire

**Figure 2:**
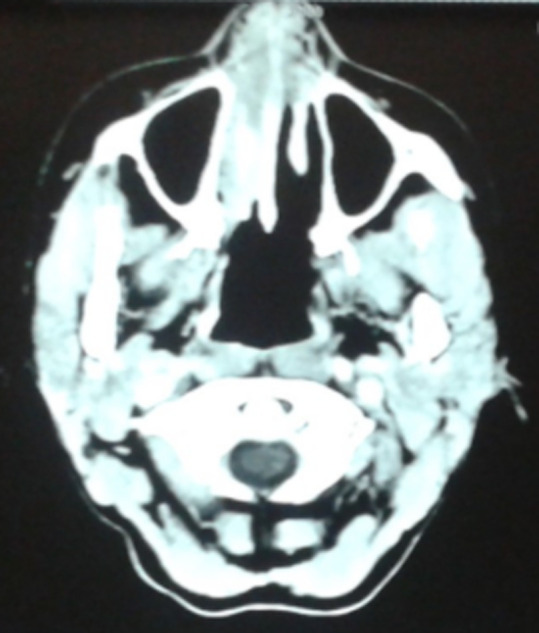
aspect scannographique en préopératoire, hyperdensité occupant les vestibules narinaires et toute la fosse nasale droite

Une femme de 33 ans nous a été adressée en février 2016 par son gynécologue. Elle se plaignait de difficultés respiratoires du fait de la présence de masses rosées dans les fosses nasales évoluant depuis plusieurs semaines, l’obligeant à respirer par la bouche. Elle est suivie en gynécologie pour une récidive de condylomes acuminés vulvaires traités avec de la Podophylline. La patiente, diagnostiquée séropositive au VIH il y’a peu et ayant débuté son traitement anti VIH (trithérapie antivirale), avoue avoir eu de multiples partenaires sexuels. Son examen physique objective un bon état général, une voix nasonnée (rhinolalie fermée) et des excroissances de couleur rosée faisant protrusion dans les deux vestibules narinaires (Figure 1), le reste de l’examen oto-rhino-laryngologie (ORL) ne retrouve aucune autre anomalie. Un scanner des cavités nasales est réalisé et met en évidence des lésions d’hyperdensité dont l’une occupe la totalité de la fosse nasale droite et l’autre se limite au vestibule narinaire et au tiers antérieur de la fosse nasale gauche (Figure 2).

**Figure 3:**
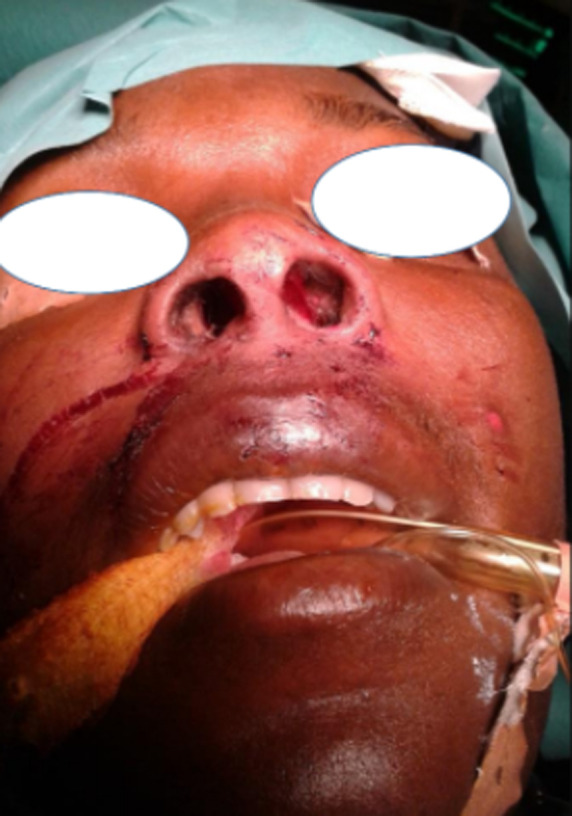
aspect post opératoire précoce, perméabilité des vestibules narinaires restaurée

**Figure 4:**
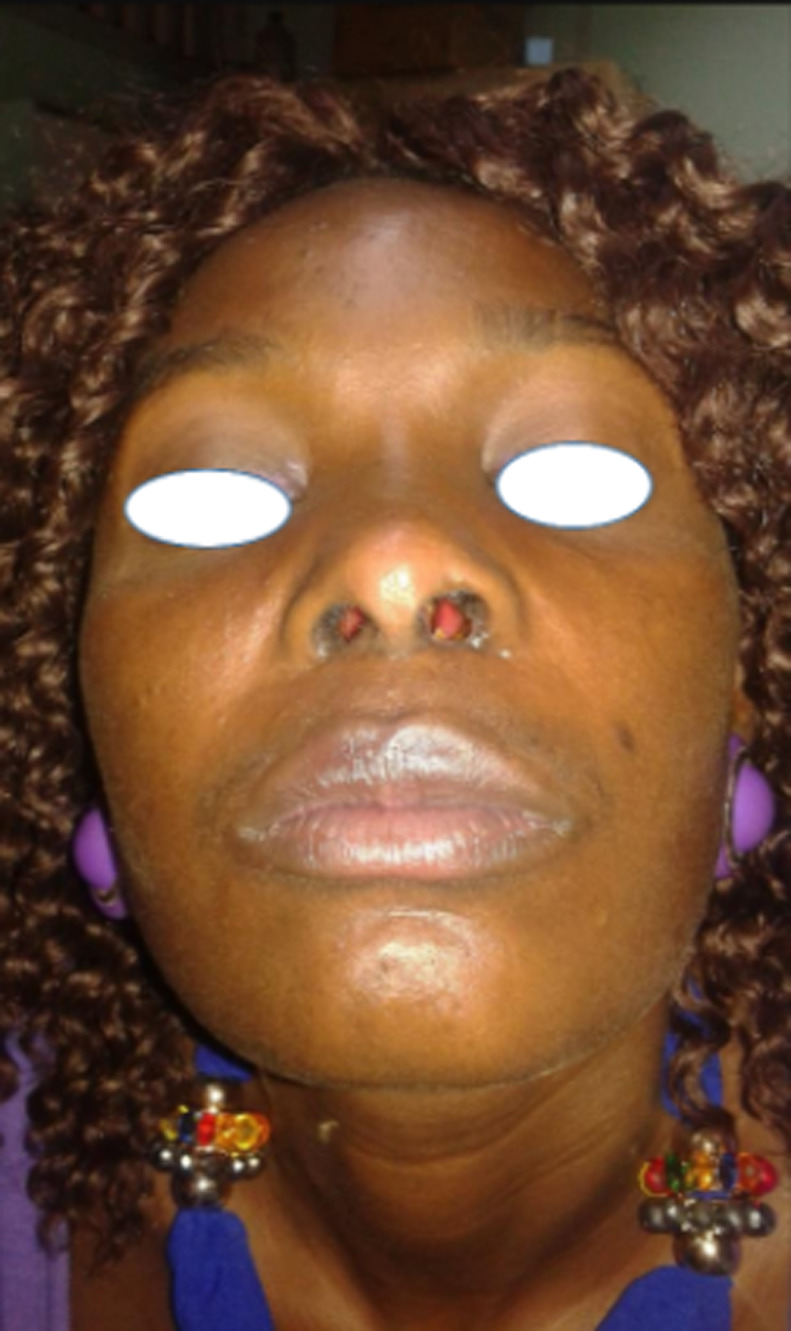
six mois post opératoire aucune récidive notée

**Figure 5:**
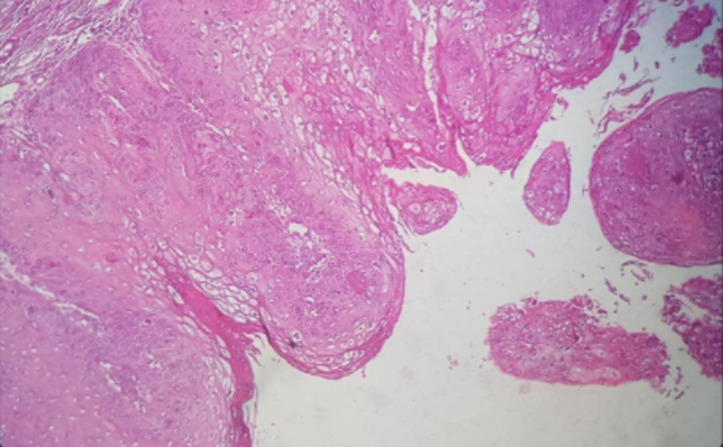
aspect microscopique des condylomes acuminés avec koilocytes et hyperkératinisation

Une exploration endoscopique des fosses nasales sous anesthésie générale est effectuée un mois plus tard et retrouve des lésions très hémorragiques, celles-ci sont excisées et leurs bases d’implantations cautérisées à la monopolaire. Ces lésions étaient greffées sur les parois des vestibules narinaires, à la jonction peau vestibulaire et muqueuse nasale avec prolongement dans les fosses nasales surtout droite. L’exploration endoscopique aux optiques rigides ne retrouve aucune autre lésion implantée dans les fosses nasale ou le cavum. La patiente est libérée du service 24 heures après sa chirurgie et des lavages des fosses nasales au sérum physiologique sont prescrits pour une dizaine de jours. Les suites postopératoires précoces et à distance sont simples (Figure 3, Figure 4). La patiente retrouve une respiration nasale satisfaisante et une voix normale. Le matériel ainsi enlevé des fosses nasales et envoyé en histopathologie parle de: «un matériel végétant, épithélium malpighien très acanthosique, hyperkératosique avec des cellules pavimenteuses montrant en surface des koilocytes; témoins d’une infection à HPV. Ces observations sont en faveur d’un condylome acuminé» (Figure 5). Le diagnostic de condylomes acuminés des fosses nasales est retenu chez cette patiente et un suivi régulier institué pour un diagnostic précoce d’éventuelles récidives. Six mois après le geste chirurgical, aucune récidive n’est à déplorer et les lésions vulvo-vaginales ont complétement disparues.

## Discussion

Les condylomes acuminés sont des lésions causées par le HPV, elles peuvent se développer aux dépens du tissu cutané et/ou des muqueuses; mais le plus souvent, à la jonction cutanéo-muqueuse [3] comme chez notre patiente où les lésions étaient greffées à la jonction peau du vestibule narinaire et muqueuse de la fosse nasale. La localisation de ces lésions au niveau des fosses nasales est exceptionnelle, seuls quelques cas ont été rapportés dans la littérature [4, 5]. La transmission du HPV est surtout sexuelle lors des contacts: peau contre peau, muqueuse contre muqueuse [6]. Ces lésions sont fréquentes chez des sujets jeunes avec une activité sexuelle à risque et de multiples partenaires [6] ce qui est le cas chez notre patiente. Le statut de séropositif au VIH est susceptible de favoriser la contamination par le HPV et partant l’apparition de condylomes acuminés. Cependant des cas de localisations multiples ont été décrits dans la littérature chez des patients plus âgés et séronégatifs au VIH, mais même chez ces derniers une forme d’immunodépression a été suspectée [7].

Les localisations orales des condylomes s’expliquent par la multiplication des rapports oro-génitaux [6]. Chez notre patiente, la localisation nasale pourrait s’expliquer par une contamination manuportée entre ses organes génitaux (vulve) et son vestibule narinaire, lors par exemple d’efforts de grattage du nez après que les doigts aient touchés la vulve. Des HPV auraient ainsi été transportés sur les doigts et ongles de la patiente et auraient été ensuite greffés sur la peau vestibulaire de la narine et la muqueuse de la fosse nasale lors d’un effort de grattage nasal. La présentation clinique de notre patiente ne diffère pas du tableau classique de tumeurs des fosses nasales; la spécificité ici est l’aspect de la lésion faisant issue dans la narine; cet aspect en crêtes de coq est assez caractéristique des condylomes acuminés et peut à ce niveau faire suspecter le diagnostic. L’analyse histologique retrouve toujours de l’hyperkératose et de l’acanthose avec des koïlocytes, cette histologie présente chez notre patiente est pathognomonique du condylome acuminé [5]. Il faut néanmoins penser à exclure en diagnostic différentiel: le papillome, le polype sneiderien, le papillome inversé et le papillome épidermoïde.

Le but du traitement est d’éradiquer les lésions et de prévenir les récidives. Le traitement peut se concevoir à travers un geste chirurgical emportant la lésion jusqu’à la couche épithéliale basale car le HPV infecte surtout les cellules de la couche basale [5]. C’est ce qui a été fait chez cette patiente avec en plus une cautérisation de la base d’implantation lésionnelle pour réduire au maximum le risque de récidive. D’autres traitements existent comme la Podophylline, le LASER CO2, la CIDOFOVIR. Cependant, en dehors de la Podophylline qui a été utilisée pour traiter les lésions vulvaires de la patiente, les autres traitements ne sont pas disponibles au Cameroun. L’évolution après un traitement bien fait est souvent favorable, cependant, il faut rester vigilant et continuer pendant plusieurs mois voire années à suivre la malade pour pouvoir réagir assez rapidement en cas de récidives.

## Conclusion

Les condylomes acuminés peuvent être localisés dans les fosses nasales. C’est une localisation rare certes mais elle est décrite dans la littérature. Dans notre contexte, devant une telle localisation, il faut réaliser un examen physique complet avec recherche d’autres localisations notamment génitales et une sérologie au VIH. Le traitement est chirurgical avec cautérisations des bases d’implantation au bistouri électrique. Un contrôle endoscopique doit être réalisé régulièrement pour dépister et prendre en charge d’éventuelles récidives.
